# Assuring Quality Health Care in the Emergency Department

**DOI:** 10.3390/healthcare3030726

**Published:** 2015-08-20

**Authors:** Susan Letvak, Denise Rhew

**Affiliations:** 1 The University of North Carolina at Greensboro, Greensboro, NC 27402, USA; 2 Emergency Services, Cone Health, Greensboro, NC 27401, USA; E-Mail: Denise.Rhew@conehealth.com

**Keywords:** quality of care, mental health, emergency department

## Abstract

The provision of quality healthcare is an international mandate. The provision of quality healthcare for mental health patients poses unique challenges. Nowhere is this challenge greater than in the emergency department. The purpose of this manuscript is to describe evidence-based initiatives for improving the quality of care of mental health patients in the emergency department. Specifically, the use of telepsychiatry and reducing provider biases will be presented.

## 1. Introduction

The provision of quality health care is an international mandate. While data are routinely collected and reported on measures such as 30-day mortality and re-admission rates for medical conditions such as heart failure and pneumonia, far less attention is paid to the quality of mental health care in hospitals, particularly in the emergency department (ED). The Centers for Disease Control and Prevention report that ED visits by patients with mental health disorders are increasing more rapidly than general ED visits [1]. Mental disorders and/or substance abuse made up one of eight emergency department visits in 2010, which is nearly 12 million visits per year [2]. Our recent economic downturn forced states to cut approximately $ 4.35 billion in public mental health spending in the period 2009–2012, the largest reduction since deinstitutionalization in the 1960s, sending many patients to the ED as their only source of healthcare [3]. Federal and state laws require all patients who present to the ED to be evaluated and stabilized, or admitted for inpatient care. However, many EDs are not equipped for provision of mental health services, and care quality is lacking. Emergency department personnel are often poorly trained in the provision of mental health services. In addition, the research literature finds quality of mental health care is also impacted by societal attitudes and healthcare provider biases, inadequate educational preparation, safety concerns and over-crowding in busy EDs, and a lack of care guidelines [4]. The purpose of this article is to describe evidence-based initiatives for improving the quality of care of mental health patients in the ED. Specifically, concerns about overcrowding and the use of telepsychiatry and reducing provider biases will be presented.

## 2. Methodology

For the purpose of this review, the problem identified was the identification of research-based and evidence-based practices to improve quality of care for mental health patients in the ED. The review was carried out via the following databases: CINAHL, EBSCO, E-Journals, Google Scholar, Medline, ProQuest, PsychInfo, PubMed, and Science Direct. To reflect the current state of the science, the search was limited to research- or evidence-based studies that were published in English between 2005 and 2015. With the assistance of a medical librarian, **s**earch terms included, “health care”, “quality of care”, “patient safety”, “mental health”, “psychiatric”, “emergency department”, “emergency room”, and “urgent care”. Because thousands of articles were identified, a Boolean search was required, utilizing the terms “quality of care or patient safety”, “mental health or psychiatric health”, and “emergency department, emergency room, or urgent care”. Additionally, studies had to be research- or evidence-based, practice-based, and peer-reviewed. Recent studies and large reviews of the literature were identified which focused on three major areas: overcrowded emergency departments, telepsychiatry, and lack of knowledge and provider bias.

## 3. Overcrowded Emergency Departments

The American College of Emergency Physicians states that the greatest threats to patient safety and quality in the ED are overcrowding and on-call specialist shortage [5]. Almost 10 years ago, the Institute of Medicine (IOM) report *Future of Emergency Care: Hospital-Based Emergency Care at the Breaking Point* warned of an overburdened emergency-care system [6]. That warning is now being felt today as EDs across the United States (US), as well as internationally, are overcrowded with high patient acuities, lengthy patient waiting times, and low patient satisfaction. Indeed, a study by Pines *et al.* [7] of ED directors found that the majority of the 15 countries outside of the US reported overcrowding and/or trends towards overcrowding. Much of the overcrowding was due to the boarding of admitted patients. Lengthy patient stays in the ED present quality of care issues for patients and significant financial burdens for hospitals [8]. A study by Nicks and Manthey [9] of one academic medical center in the US found that patients with mental illness had a longer length of stays than those without mental illness for a cost of over 2000 dollars per patient. Additionally, those with mental illness waited 3.2 times longer for an inpatient bed than those without a mental illness.

Interestingly, a study by Atzema *et al.* [10] of over 51,000 patient charts in 155 EDs in Canada found that patients with mental illness received appropriate triage scores and, indeed, when overcrowding occurred, waited less time to see a physician than those without a mental health illness. However, when the ED was not crowded, they waited a longer period of time. Important limitations to this study, however, include the fact that psychiatric medical teams existed in these facilities, mental health patients escorted by police were excluded, and there was a significant amount of missing data on wait times. It must be noted that, traditionally, EDs have relied on the model of bringing in specialized psychiatric care personnel and then discharging patients to appropriate outpatient care or admitting patients. However, there is a growing lack of psychiatrists in the US and worldwide, especially in rural and low- and middle-income countries, which will likely impact quality of care even more into the future [11].

## 4. Telepsychiatry

One evidence-based strategy to assure rapid assessment and intervention when rapid psychiatric consult is not available is through the use of telepsychiatry. The American Telemedicine Association defines telemedicine as the “use of medical information exchanged from one site to another via electronic communications to improve a patient’s clinical health status” [12]. Telepsychiatry is a form of telemedicine and is defined as “video conferencing that can provide psychiatric services to patients living in remote locations or otherwise underserved areas” [13]. Hilty *et al.* conducted a review of the literature on telepsychiatry and stated that telepsychiatry has been slower to develop in EDs compared to other specialty services, such as neurology [14]. Since this review, Seidel and Kilgus conducted a study comparing face-to-face *versus* telepsychiatry in mental health patients over 39 months in an ED [15]. The researchers found no significant differences in disposition recommendation, strength of recommendation, or diagnosis on a dangerousness scale.

Several more recent studies have demonstrated the effectiveness of telepsychiatry in assuring quality care for mental health patients in the ED setting. Southard *et al.* conducted a retrospective chart audit of mental health evaluations in a rural ED [16]. After implementation of telepsychiatry, a significant reduction in time to treatment, length of stay, and door-to-door consult time was identified. Several large hospital systems in the US are also reporting improved quality of care through ED telepsychiatry. After implementation of telepsychiatry at the Albemarle Hospital Foundation in rural North Carolina, the ED length of stay for mental health patients was more than halved and there were documented decreases in recidivism rates and involuntary commitments [17]. South Carolina has launched a state-wide initiative in 18 (rural and urban) EDs with telepsychiatry. Since March 2009, the program has served over 18,000 patients with remarkable results when compared with matched controls: a decrease in length of stay to 0.43 days from 1.35 days, a drop in admissions from 22% to 11%, and follow up rates of 46% from 16% [18].

## 5. Lack of Knowledge and Provider Bias

Quality of care of mental health patients in the ED may be compromised by both lack of knowledge and provider biases [4]. However, there is a paucity of research directly related to emergency department providers’ lack of knowledge concerning mental health patients. Jelinek *et al.* conducted a qualitative national study in Australia of the knowledge and confidence of emergency department clinicians’ management of mental health patients [19]. An analysis of 36 interviews found clinicians wanted to learn more about evidence-based strategies to provide better care. They had less confidence in effective assessment in those with high risk behaviors, providing continuity of care, dealing with dual diagnoses, prescribing medications, managing child and adolescent mental health, and balancing the ED caseload.

More research has been conducted on provider bias. Clarke *et al.* conducted a synthesis of the literature on ED staff attitudes toward mental health patients [20]. A total of 42 studies, conducted in 10 different countries, demonstrated that ED staff perceived mental health patients as a challenge. Staff feared aggressive or bizarre behavior and poor compliance in patients led to a “why bother” attitude. The researchers’ review found that most interventions were focused only on education of specific mental health disorders and not to changing attitudes. While there is a plethora of research on stigma and mental illness, and even interventions among community groups, far less research has been done on interventions with health professionals. The Mental Health Commission of Canada reports that some of the most deeply felt stigma that people experience still comes from healthcare providers [21]. This same group highlighted that physicians are often the most difficult to reach with anti-stigma messages as they do not attend programs provided by hospitals.

Knaak *et al.* conducted a synthesis of the literature on programs to decrease stigma in healthcare providers and improve outcomes [22]. A review of 22 programs found that stigma was related to pessimism about recovery, lack of skills and confidence, and lack of awareness of one’s own prejudices. The authors recommended that successful programs contain the following: include contact-based education/personal testimony, emphasize and demonstrate recovery, include multiple medium contacts (live and video), teach practical skills, dispel myths, and employ enthusiastic facilitators.

Finally, Shen *et al.* described the effect of education on Chinese medical students’ stigma towards mental illness [23]. The researchers used a pre-test, post-test design with the Attitudes towards Mental Illness (AMI) and the Attitudes Towards Psychiatry-30 (ATP-30) scales administered before and after their eight-week psychiatry internship. While attitudes towards mental health improved post-internship, the percentage of students who would consider psychiatry actually dropped from 11.4% to 6.5%.

## 6. Recommendations and Conclusions

Quality and safety of patient care of patients with mental health problems is a significant issue in healthcare, yet there is a paucity of data-based research on the topic. While research has been conducted on overcrowding, the effectiveness of telepsychiatry, and the negative impact of provider lack of knowledge and bias, there are few studies specific to quality of care for those with mental health in comparison to large studies of those with medical problems. Additionally, Plint *et al.* state that patient safety in the context of emergency medicine is a relatively new field of study [24]. Thus, it is of little surprise there is so little research specific to care quality and mental illness in emergency settings. With lack of public funding for outpatient mental health care services, mental health patients will continue to access the ED for care needs. There is a clear need for research on gaps in care quality as well as interventions to improve the care provided.

One area that clearly can be further developed and researched is the use of telepsychiatry in the emergency department. Hoffman and Kane surveyed 183 residency programs concerning education in telepsychiatry, and with a response rate of 46 programs (25%), the authors found that 45.7% are now involving telepsychiatry through either formal curriculum or informal exposure [25]. This is less than 50% of all programs in the US. Further education on how to provide telepsychiatry in psychiatry programs and research on assuring quality is needed.

Plint *et al.* made recommendations on research priorities in general patient safety for emergency medicine after conducting a four-phase consensus procedure after surveying multidisciplinary experts across North America [24]. Sixty-six research priorities were identified which were broken into four major themes: methods to identify patient safety issues, understanding human and environmental factors related to patient safety, the patient perspective, and interventions to improve patient safety. While mental health was not specifically identified, all of these priorities impact the quality of mental health care as well.

The American Hospital Association reports that there are approximately 4000 general EDs in the US and only 146 psychiatric EDs [26]. Mental health patients will continue to access emergency departments for both routine and emergency care. There should be a sense of urgency for evidence-based strategies to assure the highest quality of care for the most vulnerable of populations: those with mental illness.
